# *In-vivo* Lens Biometry Using the Novel Ultrasound Biomicroscopy

**DOI:** 10.3389/fmed.2022.777645

**Published:** 2022-02-14

**Authors:** Xiaoting Ruan, Chen Liang, Zhaoxia Xia, Xuhua Tan, Guangming Jin, Ling Jin, Zhenzhen Liu, Lixia Luo

**Affiliations:** ^1^State Key Laboratory of Ophthalmology, Zhongshan Ophthalmic Center, Sun Yat-sen University, Guangdong Provincial Key Laboratory of Ophthalmology and Visual Science, Guangdong Provincial Clinical Research Center for Ocular Diseases, Guangzhou, China; ^2^Department of Ophthalmology, The Sixth Affiliated Hospital, Sun Yat-sen University, Guangzhou, China

**Keywords:** ultrasound biomicroscopy (UBM), anterior segment, OCT, lens biometry, lens curvature, lens thickness, lens diameter

## Abstract

**Background and Aim:**

To assess the reproducibility of the novel ultrasound biomicroscopy, Insight 100 and its agreement with a swept-source optical coherence tomography, CASIA2.

**Methods:**

A total of 96 volunteers (96 eyes) were enrolled. The radius of anterior lens curvature (RAL), the radius of posterior lens curvature (RPL), lens thickness (LT), and lens diameter (LD) were measured with Insight 100 and CASIA2. A semiautomated software was used to adjust the measurement of LT (LT_S_) and LD (LD_S_) by Insight 100. Intraobserver and interobserver reproducibility of Insight 100 measurements, and the agreement of results from Insight 100 and CASIA2 were assessed with 95% limit of agreement (LoA), intraclass correlation coefficient (ICC), Pearson correlation, and linear regression.

**Results:**

For Insight 100 measurements, the intraobserver ICCs of RAL, RPL, LT_S_, and LD_S_ measurement were 0.996, 0.973, 0.936, and 0.889, and the interobserver ICCs were 0.987, 0.890, 0.974, and 0.816, respectively. There was an excellent correlation in LT measurements (*R* = 0.961, *P* < 0.001) but poor agreements in other parameters between the two devices. The LD measurements tended to be larger (95% CI: 0.768–0.928) in CASIA2 when compared with Insight 100.

**Conclusion:**

Insight 100 could obtain highly repeatable lens biometry *in vivo*. With better signal penetration, it shows promising potential in future clinical applications.

## Introduction

The measurement of lens parameters is of great importance in both research of lens function, and calculation of the intraocular lens (IOL) power (1–5). Currently, the commercially available devices providing *in vivo* lens parameters measurement include the Scheimpflug photography, anterior segment optical coherence tomography (AS-OCT) and ultrasound biomicroscopy (UBM) (6–10). Among them, the latter two could provide a full set of *in-vivo* lens biometry.

The new generation swept-source optical coherence tomography (SS-OCT), CASIA2, can provide the automatic noncontact measurement of lens parameters using a built-in program. But since the light source of CASIA2 cannot penetrate the pigmented iris, the detection area is limited (7, 8). The conventional UBM with deeper signal penetration could image the structures behind the iris where the light cannot reach. However, this contact measurement is time-consuming and less convenient (9). The newly developed very high frequency (VHF) UBM, Insight 100 using a disposable eyepiece is less invasive than the conventional UBM. It also has the advantage over the optical device of better signal penetration. With a better view of the peripheral lens behind the iris, the Insight 100 may show more potential in ensuring more accurate measurements of the lens parameters especially for patients with relative contraindications of contact examination or being contraindicated of pupil dilation.

To provide more evidence if this novel UBM device could be a potential tool for *in vivo* lens biometry, this study investigated the intraobserver and interobserver reproducibility of the Insight 100, and its agreement with the commercially available CASIA2.

## Materials and Methods

### Participants

This study was conducted at the Zhongshan Ophthalmic Center, Sun Yat-sen University, Guangzhou, China. Volunteers were consecutively recruited from the Outpatient Department from January to June 2020. Subjects with any evidence of the following conditions were excluded: 1) ocular disease besides senile cataract and refractive error; 2) history of intraocular surgery; and 3) inability to cooperate with the test, or poor fixation resulting in low image quality. The study was approved by the ethics committee of Zhongshan Ophthalmic Center and was performed following the tenets of the Declaration of Helsinki. Written informed consents were obtained from all participants.

### Anterior Segment Scanning

Anterior segment scanning was performed with SS-OCT, CASIA2 (Tomey Corporation, Nagoya, Japan) and the UBM, Insight 100 (ArcScan Incorporation, Morrison, Colorado, USA). These two devices were operated independently by two experienced operators (XT-R and C-L) in random sequence. The operators were masked to results of one another. Neither mydriatic nor miotic drops were applied before the test, to avoid any accommodation stimulus. Scanning by the two devices was performed in the same room and under the same lighting conditions. Fixation target is consistent during measurements in CASIA2 and Insight 100.

### CAISA2 Scanning

The CASIA2 uses a swept-source laser with a wavelength of 1,310 nm at a velocity of 50,000 A-scan/s. The axial and transverse resolutions are 10 μm or less and 30 μm or less, respectively. The participants were seated and asked to fixate on the external lights during the examination. Lens biometric parameters including radius of anterior lens surface curvature (RAL), the radius of posterior lens surface curvature (RPL), lens thickness (LT), and lens diameter (LD) were automatically measured by the built-in software (Version 3G.1). The CASIA2 measurements of lens biometry were aligned along the visual axis and performed using the 16-scan “lens biometry” mode. Then the measurement results were automatically generated by the “Lens Analysis” module in “Lens Biometry” mode. After the measurement, the operator can check the clarity and fixation of all images in the preview, after which the qualified measurements were selected for analysis. The 2D analysis results of 0–180 degree in “Len Biometry” mode was used for the comparison with UBM.

### Insight 100 Scanning

The Insight 100 examination for lens biometry was performed using the “capsule” mode with a broadband 20–60 MHz VHF ultrasound transducer. This system has a tissue penetration depth of 15 mm. The entire anterior segment is presented in a single image with an axial resolution of 35 μm and lateral resolution of 65 μm. The scan is angular (2 scans/s); the image area is adjustable by the user up to 70 degrees and 22 mm. The ultrasound velocity in this study was set at 1,640 m/s for the lens capsule. The capsular bag was examined on the axial horizontal section (transverse diameter passing through the corneal apex from 9 to 3 o'clock). During UBM scanning, the participants were seated with the chin and forehead placed into a headrest. A soft rimmed eye-cup was placed in the eye to be examined, with a soft membrane separating the eye from the transducer and scanning chamber, which was filled with distilled water. Participants fixated with the fellow eye on a narrow fixation target to ensure that the corneal vertex was coaxial with the infrared camera and the scanning rotation center. The examiner clicked on the video feed of the eye to adjust the system to be centered on the corneal reflex. The automated centration algorithm used information from horizontal and vertical scans to find the corneal vertex. The measurement achieved quality control by built-in software for statistical analysis.

The scenario of the Insight 100 examination is illustrated in Supplementary Figure S1. The acquisition with the qualified fixation was selected for further analysis of crystalline lens biometry.

### Manual Measurement Using Insight 100 Images

Two ophthalmologists (XT-R and C-L) independently measured the RAL, RPL, LT, and LD in the images by using the built-in manual caliper tool of UBM. The built-in manual caliper tool can generate a fitting arc through three points. The RAL was determined as follows: the first two points were defined as where the anterior lens capsule intersects with the iris, and the third point was the apex of the anterior lens capsule. The RPL is determined similarly to the above three points. The fitting curve could be adjusted *via* moving these three points for the best fit. The LT was defined as the distance between apexes of the anterior and posterior lens surfaces. The LD was defined as the distance between the intersections of the anterior and posterior lens surface fitting curves. Each selected scan was measured by one ophthalmologist repeating three times.

### Semiautomated Measurement Using Insight 100 Images

**Figure 1:**
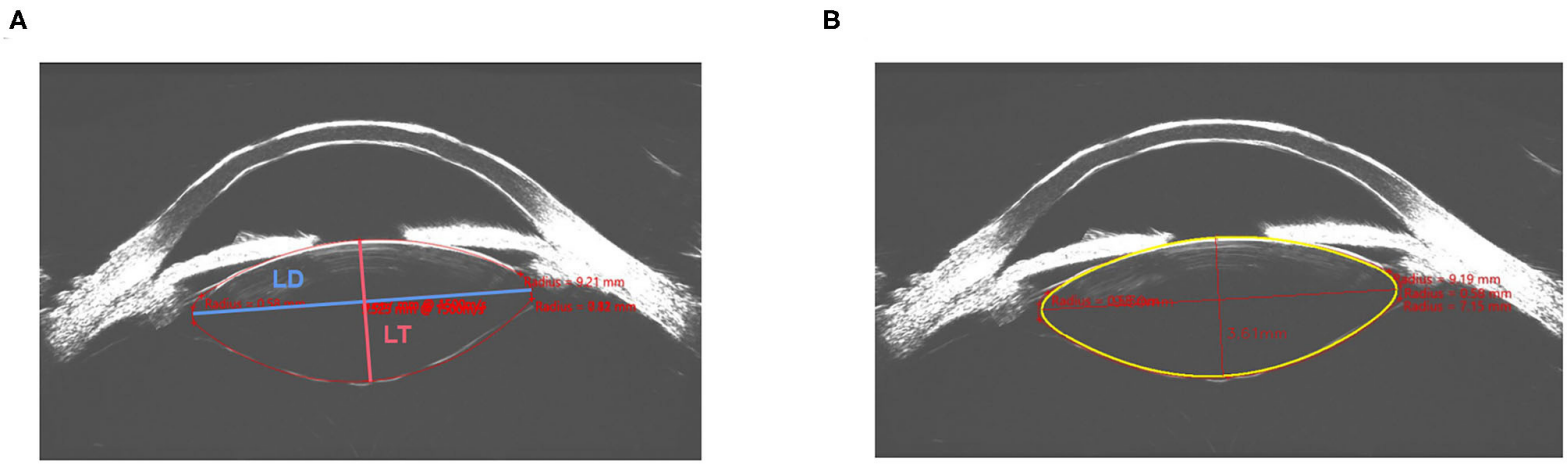
Manual measurement **(A)** and semiautomated measurement **(B)** of the lens diameter (LD) and lens thickness (LT) in the same image obtained by Insight 100.

After input of the contour line and curvature of the anterior and posterior lens surface, the customized developed software can automatically process further segmentation and fitting based on the input data. After adjustment, more accurate contour lines of the anterior, posterior, left, and right surface of the lens were obtained generating semiautomated measurements of the lens thickness and lens diameter (LD_S_ and LT_S_). Figure 1 shows the manual measurement and semiautomated measurement of the LD_S_ and LT_S_ in the Insight 100 scan of the same eye.

### Statistical Analysis

The sample size was calculated based on the interobserver agreement of Insight 100, which was assessed using intraclass correlation (ICC). A sample of 93 subjects who were each measured 2 times was necessary to estimate the ICC of 0.8 and a two-sided 95% CI with a width of 0.100, using a two-way random-effects ANOVA model. The PASS 16.0 (NCSS, LLC, Kaysville, Utah, USA) was used to calculate the sample size.

With the binocular parameters comparable in subjects, the right eyes were chosen for analysis. All continuous variables were expressed as mean ± SD. The ICCs and the 95% limit of agreement (LoA) were used to assess intraobserver and interobserver reproducibility of the manual measurements of RAL, RPL, and semiautomated measurement of LT_S_, LD_S_ using Insight 100 scans. The ICC estimates and their 95% CIs were calculated based on an absolute-agreement, two-way random-effects model. For each eye, the comparison was made between the average lens parameters of three repeated Insight 100 scans and the 2D result in a horizontal position from CASIA2. The 95% LoA, the ICC (two-way random model), Pearson correlation analysis, and Bland–Altman plot were used for comparing the agreement between measurements from the Insight 100 and the CASIA2. The linear regression was used to evaluate the conversion of lens biometry between the Insight 100 and the CASIA2. All statistical analyses were performed using SPSS statistical software (SPSS Statistics version 22.0; IBM Corp., Armonk, NY, USA). Statistical significance was defined as *P* < 0.05.

## Results

In this study, 96 eyes from 96 volunteers (40 male and 56 female) were included. The mean age of all participants was 34.42 ± 11.19 years old, ranging from 23 to 74 years old. Baseline characteristics of the participants and the pipeline of the study were demonstrated in Supplementary Figure S2 and Supplementary Table S1. All participants had completed both CASIA2 and UBM tests.

Two examiners performed measurements for all participants using Insight 100 scans. There were excellent intraobserver repeatabilities for manual measurements of RAL, RPL, and semiautomated measurements of LT_S_ and LD_S_ (ICC: 0.996, 0.973, 0.936, and 0.889, respectively). And as shown in Table 1, the interobserver reproducibilities were excellent in measuring RAL and LT_S_ (ICC: 0.987, 0.974, respectively) and good in measuring RPL and LD_S_ (ICC: 0.890, 0.816, respectively).

**Table 1:** Interobserver reproducibility of lens biometry measurements with Insight 100.

		**Mean (mm^**3**^)**	**SD (mm^**3**^)**	**Mean difference (95% CI)**	***P* value**	**Interobserver reproducibility**	
						**95%LoA**	**ICC**
RAL	Observer 1	10.308	1.530	−0.079(−0.128 to −0.030)	0.002*	−0.392 to 0.549	0.987
	Observer 2	10.229	1.562				
RPL	Observer 1	6.388	0.752	0.142(0.077 to 0.207)	<0.001*	−0.770 to 0.486	0.890
	Observer 2	6.530	0.724				
LT_S_	Observer 1	3.564	0.345	0.022(0.006 to 0.037)	0.006*	−0.170 to 0.127	0.974
	Observer 2	3.586	0.342				
LD_S_	Observer 1	9.032	0.387	0.115(0.074 to 0.157)	<0.001*	−0.516 to 0.286	0.816
	Observer 2	9.148	0.368				

*RAL, radius of anterior lens surface curvature; RPL, radius of posterior lens curvature; LT, lens thickness; LD, lens diameter; LT_S_, lens thickness in semiautomated measurement; LD_S_, lens diameter in semiautomated measurement; LoA, the limit of agreement; ICC, intraclass correlation coefficient.*

Bold*: Statistically significant difference (P < 0.05).

**Figure 2:**
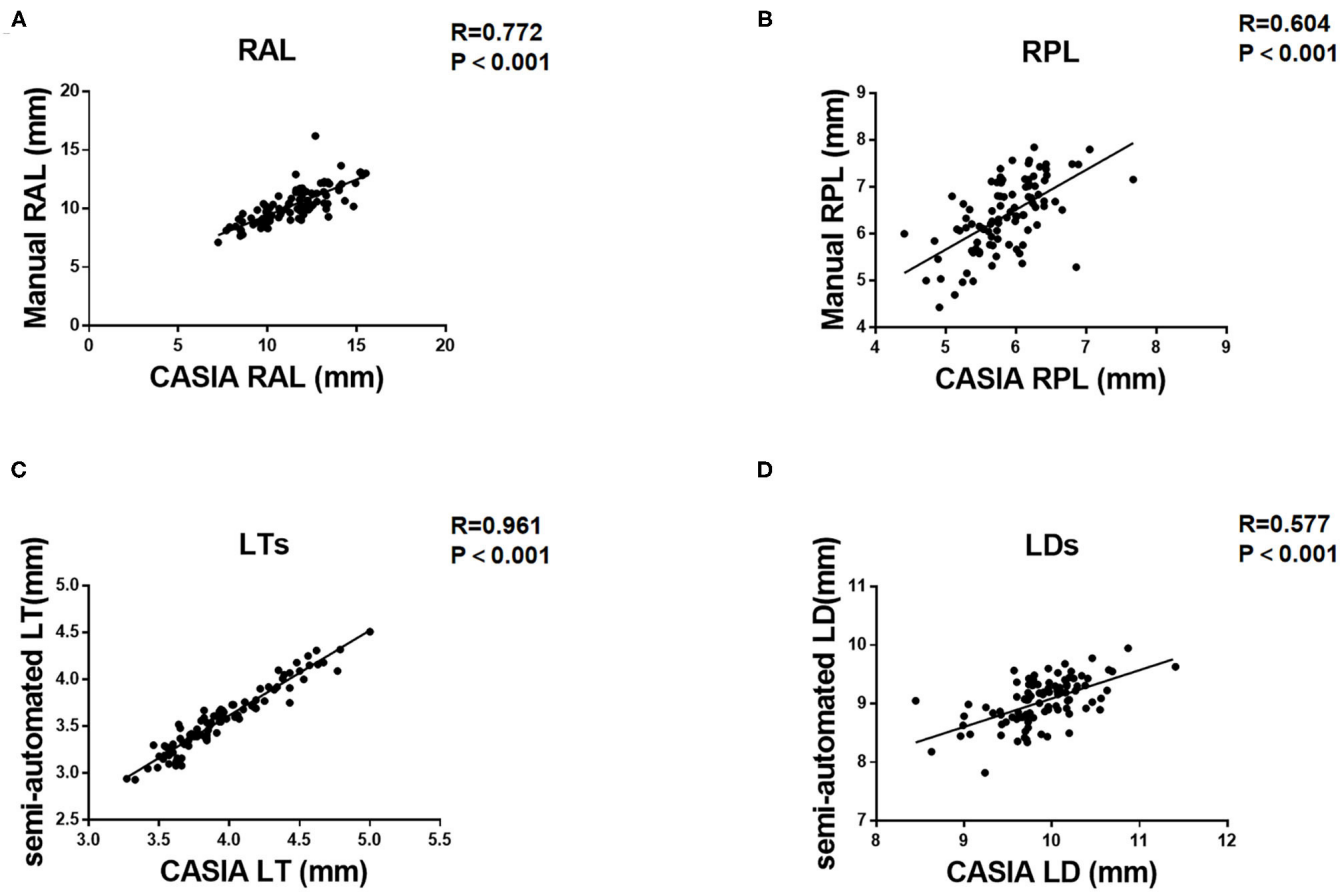
Pearson correlation coefficient (*R*) between Insight 100 and CASIA2 in measuring RAL **(A)**, RPL **(B)**, LT_S_ **(C)**, and LD_S_ **(D)**.

**Figure 3:**
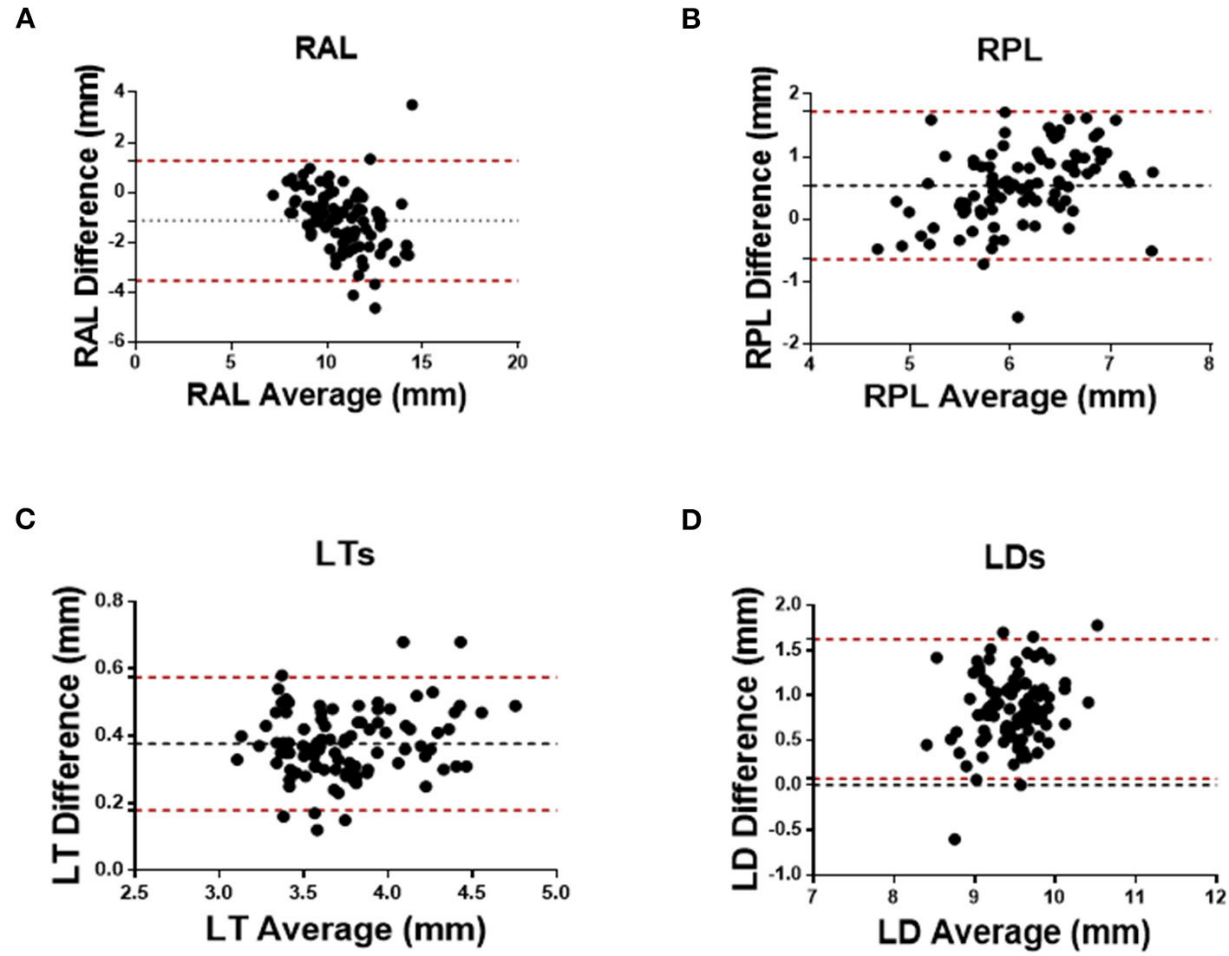
Bland–Altman analysis between Insight 100 and CASIA2 in measuring RAL **(A)**, RPL **(B)**, LT_S_ **(C)**, and LD_S_ **(D)**.

As for the agreement of Insight 100 and CASIA2, the Pearson correlation coefficient (*R*) in measuring RAL, RPL, LT, and LD was 0.772 (*P* < 0.001), 0.604 (*P* < 0.001), 0.961 (*P* < 0.001), and 0.577 (*P* < 0.001), respectively (Figure 2). According to the Bland–Altman analysis shown in Figure 3, the CASIA2 gave larger measures in lens thickness and lens diameter than that from the semiautomated measurements from Insight 100. And these differences tended to be consistent across the measurement ranges. The linear regression equations for measurement conversion were as follows (Y represented measurement using the Insight 100, X represented measurement using the CASIA2):


$$\begin{array}{lll} \text{RAL}:{\text{Y}}_{\text{Insight}100} & = & 0.6126^{*}{\text{X}}_{\text{CASIA}2}+3.300\left(\text{P}<\;0.001\right)\\ \text{RPL}:{\text{Y}}_{\text{Insight}100} & = & 0.8503^{*}{\text{X}}_{\text{CASIA}2}+1.415\left(\text{P}<\;0.001\right)\\ \text{LT}:{\text{Y}}_{\text{Insight}100} & = & 0.9076^{*}{\text{X}}_{\text{CASIA}2}-0.01326\left(\text{P}<\;0.001\right)\\ \text{LD}:{\text{Y}}_{\text{Insight}100} & = & 0.4845^{*}{\text{X}}_{\text{CASIA}2}+4.245\left(\text{P}<\;0.001\right) \end{array}$$

Table 2 shows the comparison of lens biometry measured with the CASIA2 and the Insight 100. The 95% LoA between the CASIA2 and the Insight 100 was −3.537 to 1.271 mm (RAL), −0.646 to 1.724 mm (RPL), 0.179–0.575 mm (LT), 0.072–1.623 mm (LD); and the ICCs were 0.622, 0.427, 0.615, and 0.191 for the above-mentioned parameters, respectively.

**Table 2:** Comparison of lens biometry measured with CASIA2 and Insight 100.

	**CASIA2 (Mean ±SD, mm)**	**Insight 100 (Mean ±SD, mm)**	**Mean Difference (95% CI)**	***P value***	**95% LoA**	***R***	**ICC**
RAL	11.441, 1.929	10.308, 1.530	1.133(0.884 to 1.381)	<0.001*	−3.537 to 1.271	0.772	0.622
RPL	5.849, 0.534	6.388, 0.752	−0.539(-0.661 to−0.417)	<0.001*	−0.646 to 1.724	0.604	0.427
LT_S_	3.941, 0.366	3.564, 0.345	0.377(0.357 to 0.398)	<0.001*	0.179 to 0.575	0.961	0.615
LD_S_	9.880, 0.461	9.032, 0.387	0.848(0.768 to 0.928)	<0.001*	0.072 to 1.623	0.577	0.191

RAL, radius of anterior lens surface curvature; RPL, radius of posterior lens curvature; LT_S_, lens thickness in semiautomated measurement; LD_S_, lens diameter in semiautomated measurement; LoA, the limit of agreement; ICC, intraclass correlation coefficient; Bold^*^: Statistically significant difference (P < 0.05).

## Discussion

As a less invasive novel VHF UBM, Insight 100 showed excellent intraobserver and interobserver reproducibility in measuring RAL, RPL, LT, and LD, which has not been reported by previous studies. There was an excellent agreement between Insight 100 and CASIA2 in measuring lens thickness, with a relatively poor agreement in the measurement of RAL, RPL, and lens diameter. And measurements of lens thickness and diameters from Insight 100 tended to be smaller when compared with those from CASIA2.

By now, there is no golden standard of the *in vivo* lens biometry, that comparisons among different devices are still inconclusive if which one would be the most accurate. Several studies have been conducted to investigate the performance of different devices, which are summarized in Table 3. In general, results from CASIA2 were proved to be reproducible, with better performance in younger individuals. The RAL measurements could be interchangeable between CASIA2 and Scheimpflug imaging. While the measurement of the posterior lens was shown to be less stable, which could be affected by mild cataract. Also, the correlation was poor in measuring the posterior lens between CASIA2 and biometry with better penetration, for example, the ultrasound-based Insight 100.

**Table 3:** Comparison of lens parameters measurement with different devices from the current vs. previous studies.

**Study**	**Participants**		**Device**	**Parameters**							
	**Number (subjects, eyes)**	**Age (mean, range)**		**RAL**		**RPL**		**LT**		**LD**	
				**Mean ±SD (mm)**	**ICC**	**Mean ±SD (mm)**	**ICC**	**Mean ±SD (mm)**	**ICC**	**Mean ±SD (mm)**	**ICC**
Current study	96, 96	34.42 (23-74)	Insight 100	10.308 ± 1.530	0.987	6.388 ± 0.752	0.890	3.564 ± 0.345	0.974	9.032 ± 0.387	0.816
			CASIA2	11.441 ± 1.929	N/A	5.849 ± 0.534	N/A	3.941 ± 0.366	N/A	9.880 ± 0.461	N/A
Fukuda (8)	50, 50	30.1	CASIA2	12.16 ± 1.60	0.996	6.09 ± 0.40	0.937	3.77 ± 0.20	0.998	10.03 ± 0.32	0.924
	78, 135	72.1	CASIA2	9.66 ± 1.41	0.993	5.81 ± 0.56	0.674	4.56 ± 0.39	0.995	10.14 ± 0.61	0.827
Shoji et al. (7)	30, 30	35.6	CASIA2	10.4 ± 1.5	0.857 0.914	5.8 ± 0.4	0.879 0.933	3.9 ± 0.3	0.987 0.992	N/A	N/A
Liu et al. (6)	59, 59	19-87	CASIA2	10.38 ± 1.78	N/A	N/A	N/A	N/A	N/A	N/A	N/A
			Scheimpflug	8.12 ± 1.40	0.996 0.947	N/A	N/A	N/A	N/A	N/A	N/A
Li et al. (10)	25, 25	32.92 (22-66)	Insight 100	11.90 ± 2.42	N/A	6.37 ± 1.66	N/A	3.50 ± 0.35	N/A	9.76 ± 0.54	N/A
			CASIA2	11.88 ± 1.84	N/A	5.74 ± 0.51	N/A	3.84 ± 0.38	N/A	9.74 ± 0.43	N/A

*RAL, radius of anterior lens surface curvature; RPL, radius of posterior lens curvature; LT, lens thickness; LD, lens diameter; ICC, intraclass correlation coefficient*.

Using different imaging principles, the measurements of Insight 100 and CASIA2 would be affected by different factors. The optical CASIA2 requires correction of optical distortion from the cornea and aqueous humor when measuring the anterior lens surface; and measurement of the posterior lens surface would be further affected by the heterogeneity of refractive indices of the lens (9). Insight 100 is an ultrasound device with better signal penetration. The difference in imaging principles and signal penetration may explain the poor agreement in anterior lens surface measurement, and the even poorer agreement in measuring posterior lens surface. On the other hand, while inconsistent reproducibilities in measuring posterior lens surface by CASIA2 have been reported, the Insight 100 showed excellent intraobserver repeatability and good interobserver reproducibility in measuring RPL according to the current study (7, 8).

There have been limited reports on the measurement of lens diameter, which is an important assessment for postoperative IOL stability (3, 11). CASIA2 using infrared light unable to penetrate the iris can only detect the lens limited to the pupil area. And the measurement of lens diameter in CASIA2 is based on a simulated image with sharp peripheral lens angles, which surely does not match with the real shape of the human lens. The Insight 100 can detect the area behind the iris, providing a better view of the peripheral lens. By further processing with the semiautomated software, the adjusted peripheral contour lines present a more accurate ellipsoid-shape lens rather than a spindle-shaped one in CASIA2. This would explain why the measurements of lens diameter in Insight 100 were smaller than those in CASIA2. On the other hand, the semiautomated software showing good reproducibility in measuring lens diameter may be promising in clinical assessment, though further clinical studies are still needed.

While measurement of the lens surface curvature and lens diameter would be affected by the lens periphery, the measurement of lens thickness could be achieved based on the central part of the lens even with limited visualization of the periphery. This could explain the good agreement of Insight 100 with CASIA2. Moreover, high reproducibilities of lens thickness measurement in different devices were also reported previously (7–9). The lens thickness is proved to be correlated with postoperative IOLs rotational stability (4). Therefore, Insight 100 also showed potential in future clinical assessment.

When compared with previous studies, the sample size of our study is relatively large (96 eyes from 96 subjects, larger than that in the study by Liu et al. (6), Shoji et al. (7), and Li et al. (10), but smaller than that in the study by Fukuda et al. (8), with a wide age range (23–74 years old). To the best of our knowledge, this study is the first study to report the reproducibility of the novel UBM, Insight 100 in measurements of lens biometry *in vivo*. Results suggested that Insight 100 could provide highly repeatable measurements of the lens parameters *in vivo*. Based on a simulated image that was closer to the real lens shape, stable measurement of lens diameter could be obtained after further processing with semiautomated software. Agreement between Insight 100 and CASIA2 was good in measuring lens thickness, but poor in anterior and posterior lens curvature and lens diameter measurement. With better visualization of the peripheral lens, Insight 100 would be promising as an *in vivo* biometry in real clinical practice.

The limitation of the Insight 100 should be considered for further clinical application of the device. First, Insight 100 requires a manual caliper while CAISA2 can calculate the result automatically with a built-in program. However, the intraobserver and interobserver reproducibility of Insight 100 were high among all lens parameters, indicating Insight 100 could provide repeatable results. Second, a qualified evaluation of the accommodation during the Insight 100 test was unavailable. In this study, only healthy individuals were included. With higher signal penetration depth superior to the optical device, the UBM would be more preferable in patients with refractive media opacity, for example, corneal scar. Further study including subjects with other ocular diseases (e.g., corneal opacity, cataract of various stages) and subgroup analysis would be investigated to better evaluate the effectiveness and clinical potential of Insight 100.

## Conclusion

The novel VHF UBM, Insight 100 could provide repeatable full-set *in-vivo* lens parameter measurements. It showed good agreement with the commercially available SS-OCT in measuring lens thickness, but poor agreement in anterior and posterior lens curvature, and lens diameter. With deeper signal penetration, the Insight 100 has the advantage of providing a more comprehensive analysis of the lens parameter, especially in the posterior and peripheral lens. In general, the Insight 100 could be a promising tool in measuring lens parameters *in vivo*, with potential roles in preoperative IOLs selection and postoperative assessment.

## Data Availability Statement

The original contributions presented in the study are included in the article/Supplementary Material, further inquiries can be directed to the corresponding authors.

## Ethics Statement

The studies involving human participants were reviewed and approved by the Ethics Committee of Zhongshan Ophthalmic Center. The patients/participants provided their written informed consent to participate in this study. Written informed consent was obtained from the individual(s) for the publication of any potentially identifiable images or data included in this article.

## Author Contributions

ZL and LL: study concept and design and study supervision. XR, CL, and ZL: drafting of the manuscript. LL, ZL, and ZX: critical revision of the manuscript. XT and GJ: statistical analysis. All authors have full access to all the data in the study and take responsibility for the integrity of the data and the accuracy of the data analysis, acquisition, analysis, or interpretation of data, completed and submitted the ICMJE form for disclosure of potential conflicts of interest. All authors contributed to the article and approved the submitted version.

## Funding

This study was supported by the National Natural Science Foundation of China (81873675 and 81770905) and the Construction Project of High-Level Hospitals in Guangdong Province (303020102).

## Conflict of Interest

The authors declare that the research was conducted in the absence of any commercial or financial relationships that could be construed as a potential conflict of interest.

## Publisher's Note

All claims expressed in this article are solely those of the authors and do not necessarily represent those of their affiliated organizations, or those of the publisher, the editors and the reviewers. Any product that may be evaluated in this article, or claim that may be made by its manufacturer, is not guaranteed or endorsed by the publisher.
