# Supplementary material for: In-vivo Lens Biometry Using the Novel Ultrasound Biomicroscopy
Source: Front Med (Lausanne). 2022 Feb 14;9:777645. doi: 10.3389/fmed.2022.777645 (PMC8882853; doi:10.3389/fmed.2022.777645)
Supplement: Supplementary file 1 [file Table_1.docx]

**Table S1. Baseline characteristics of the participants**

| **Characteristics** | **Value**  (N=96 persons, 96 eyes) |
| --- | --- |
| **Age** (Mean±SD, years) | 34.42±11.19 |
| 20-30 years, n (%) | 47(48.96) |
| 30-40 years, n (%) | 23(23.96) |
| ＞40years, n (%) | 26(27.08) |
| **Gender** |  |
| Male, n (%) | 40(41.67) |
| Female, n (%) | 56(58.33) |

SD, Standard Deviation; N, number
