# Supplementary figures and images for: In-vivo Lens Biometry Using the Novel Ultrasound Biomicroscopy
Source: Front Med (Lausanne). 2022 Feb 14;9:777645. doi: 10.3389/fmed.2022.777645 (PMC8882853; doi:10.3389/fmed.2022.777645)

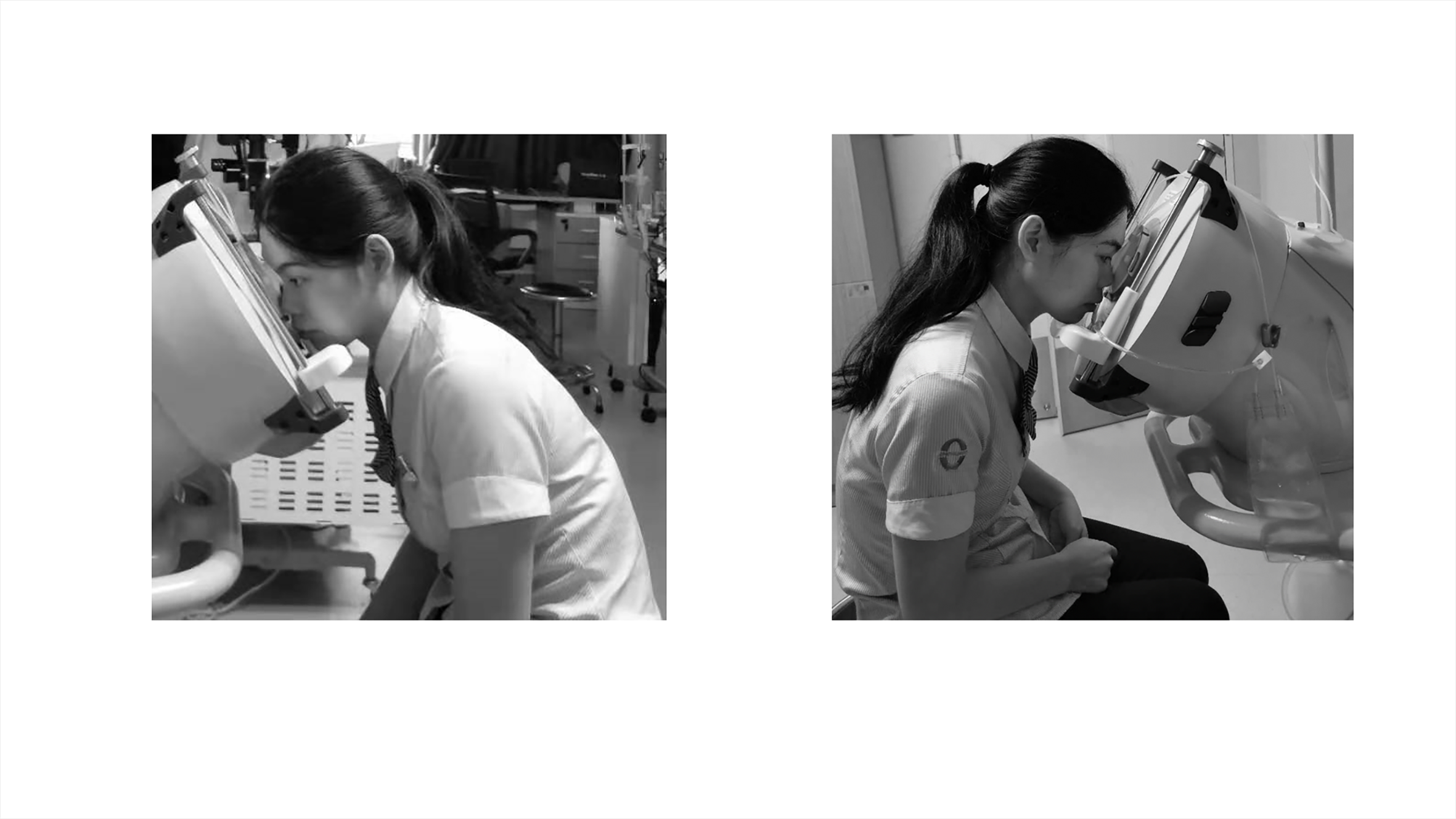

Supplement: Supplementary file 2 [file Image_1.TIF]

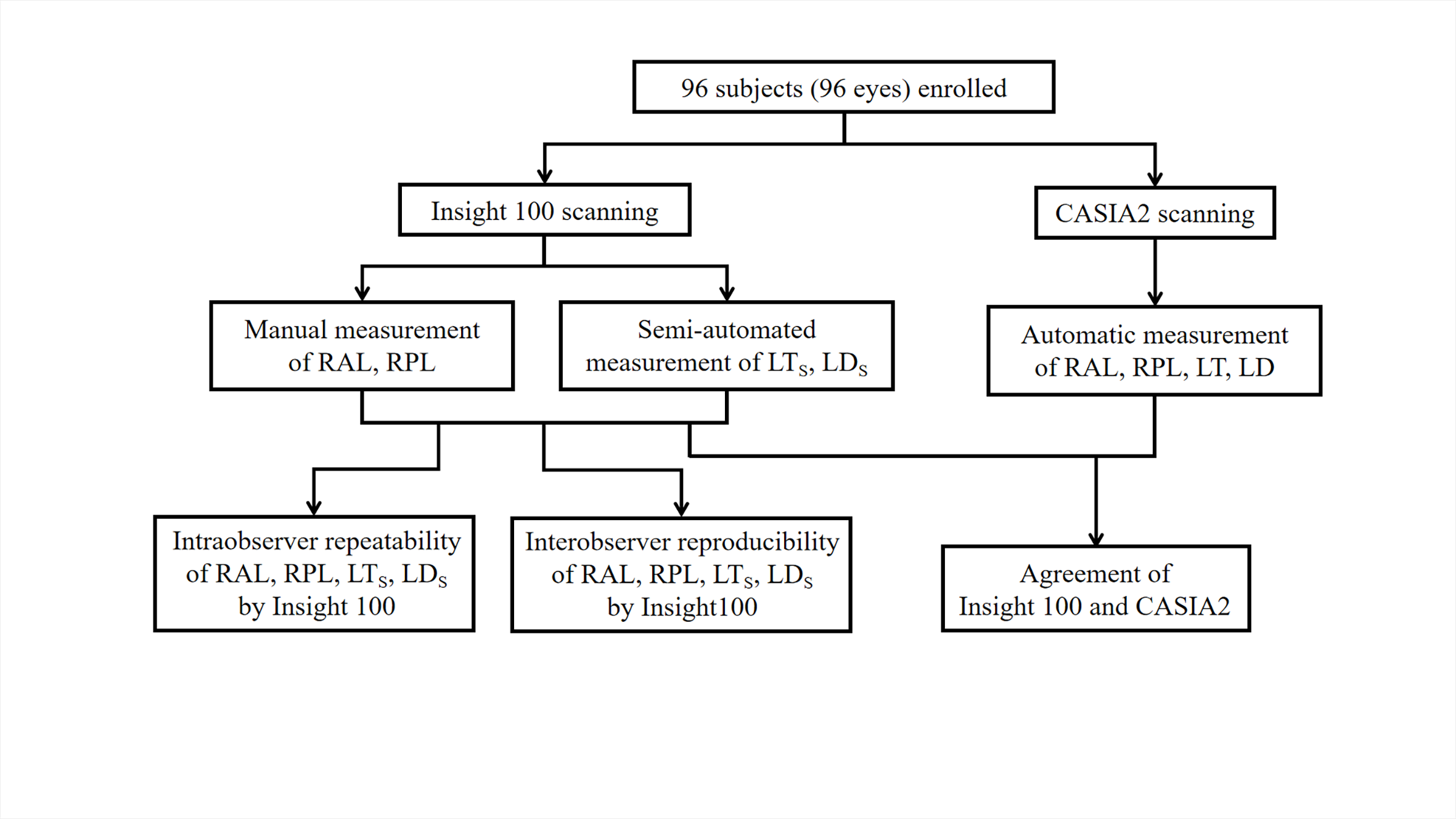

Supplement: Supplementary file 3 [file Image_2.TIF]
